# Assessment of Cardiovascular Disease Risk and Therapeutic Patterns among Urban Black Rheumatoid Arthritis Patients

**DOI:** 10.3390/medsci7020031

**Published:** 2019-02-20

**Authors:** Isabel M. McFarlane, Su Yien Zhaz Leon, Manjeet S. Bhamra, Aaliya Burza, Stephen Anthony Waite, Milena Rodriguez Alvarez, Kristaq Koci, Nicholas Taklalsingh, Ian Kaplan, Joshy Pathiparampil, Naureen Kabani, Elsie Watler, Cristina S. Sorrento, Mosab Frefer, Vytas Vaitkus, Jason Green, Keron Matthew, Fray Arroyo-Mercado, Helen Lyo, Faisal Soliman, Randolph A. Sanchez, Felix M. Reyes, David J. Ozeri, Veena Dronamraju, Michael Trevisonno, Christon Grant, Guerrier Clerger, Khabbab Amin, Latoya Freeman, Makeda Dawkins, Diana Lenis Lopez, Jonathan Smerling, Irfan Gondal, Elaine Dellinger, Karen Paltoo, Hina Bhat, Srinivas Kolla

**Affiliations:** 1 Department of Medicine, Division of Rheumatology SUNY Downstate Medical Center/Health + Hospitals Kings County, Brooklyn, NY 11201, USA; Manjeet.Bhamra@downstate.edu (M.S.B.); milena.rodriguezalvarez@downstate.edu (M.R.A.); Kristaq.Koci@downstate.edu (K.K.); Nicholas.Taklalsingh@downstate.edu (N.T.); Ian.Kaplan@downstate.edu (I.K.); Joshy.Pathiparampil@downstate.edu (J.P.); Naureen.Kabani@downstate.edu (N.K.); Elsie.Watler@nychhc.org (E.W.); Cristina.Sorrento@downstate.edu (C.S.S.); Mosab.Frefer@downstate.edu (M.F.); Vytas.Vaitkus@downstate.edu (V.V.); Jason.Green@downstate.edu (J.G.); Keron.Matthew@downstate.edu (K.M.); Fray.Arroyo-Mercado@downstate.edu (F.A.-M.); Helen.Lyo@downstate.edu (H.L.); veena.dronamraju@downstate.edu (V.D.); Michael.Trevisonno@downstate.edu (M.T.); Christon.Grant@downstate.edu (C.G.); Guerrier.Clerger@downstate.edu (G.C.); khabbab.amin@downstate.edu (K.A.); Latoya.Freeman@downstate.edu (L.F.); Makeda.Dawkins@downstate.edu (M.D.); Diana.Lenis@downstate.edu (D.L.L.); Jonathan.Smerling@downstate.edu (J.S.); Irfan.Gondal@downstate.edu (I.G.); Elaine.Dellinger@downstate.edu (E.D.); Karen.Paltoo@downstate.edu (K.P.); Hina.Bhat@downstate.edu (H.B.); 2 Samaritan Medical Center Department of Rheumatology, Watertown, NY 13601, USA; szhaz@shsny.com; 3 Department of Medicine, Division of Pulmonary and Critical Care State, SUNY Downstate Medical Center/Health + Hospitals Kings County, Brooklyn, NY 11201, USA; Aaliya.Burza@downstate.edu; 4 Department of Radiology SUNY Downstate Medical Center/Health + Hospitals Kings County, Brooklyn, NY 11201, USA; Stephen.Waite@downstate.edu (S.A.W.); Srinivas.Kolla@downstate.edu (S.K.); 5 Department of Geriatrics, New York Presbyterian Brooklyn Methodist Hospital, Brooklyn, NY 11201, USA; fas9051@nyp.org; 6 Department of Rheumatology, Hahnemann Hospital, Philadelphia, PA 19019, USA; Randysanchez2011@gmail.com; 7 Department of Family and Social Medicine, Montefiore Medical Center Albert Einstein College of Medicine, Bronx, NY 10468, USA; freyesv@montefiore.org; 8 Sheba Medical Center, Tel Aviv 6100000, Israel; david.ozeri@sheba.health.gov.il

**Keywords:** rheumatoid arthritis, traditional risk factors, rheumatoid arthritis specific risk factors, seropositive rheumatoid arthritis, erosive disease, extra-articular manifestations, cardiovascular outcomes, therapeutic patterns

## Abstract

Rheumatoid arthritis (RA) patients have nearly twice the risk of cardiovascular disease (CVD) compared to the general population. We aimed to assess, in a predominantly Black population, the prevalence of traditional and RA-specific CVD risk factors and therapeutic patterns. Utilizing ICD codes, we identified 503 RA patients ≥18 years old who were seen from 2010 to 2017. Of them, 88.5% were Black, 87.9% were women and 29.4% were smokers. CVD risk factors (obesity, diabetes, hypertension, dyslipidemia) were higher than in previously reported White RA cohorts. Eighty-seven percent of the patients had at least one traditional CVD risk factor, 37% had three or more traditional CVD risk factors and 58% had RA-specific risk factors (seropositive RA, >10 years of disease, joint erosions, elevated inflammatory markers, extra-articular disease, body mass index (BMI) < 20). CV outcomes (coronary artery disease/myocardial infarction, heart failure, atrial fibrillation and stroke) were comparable to published reports. Higher steroid use, which increases CVD risk, and lesser utilization of biologics (decrease CV risk) were also observed. Our Black RA cohort had higher rates of traditional CVD risk factors, in addition to chronic inflammation from aggressive RA, which places our patients at a higher risk for CVD outcomes, calling for revised risk stratification strategies and effective interventions to address comorbidities in this vulnerable population.

## 1. Introduction

Rheumatoid arthritis (RA) is a chronic inflammatory condition that affects about 1% of the adult population, with an annual incidence rate of 40 new cases per 100,000 persons [1,2]. The pathophysiology of RA involves a complex interplay of environmental and genetic factors, leading to chronic inflammation that erodes articular cartilage and bone. Thus, RA presents with joint pain, swelling and stiffness as inflammation and synovitis develops [1]. Chronic inflammation can impact other organ systems, leading to extra-articular manifestations including rheumatoid nodules, vasculitis, interstitial lung disease and premature death [3]. About half of all RA-related deaths can be attributed to CVD, as inflammation has been shown to play a key role at every stage of atherogenesis, further strengthening the association between cardiovascular disease (CVD) and RA [3,4,5,6,7]. The degree of CVD involvement in RA has been shown to correlate with the degree of systemic inflammation [8,9,10]. In support of this association, disease-modifying, anti-rheumatic drugs (DMARDs) and biologics in RA have been shown to confer protection from CVD-related events [11,12,13,14]. Recently, epidemiologic data indicates that African American patients have higher rates of CVD associated with connective tissue disease compared with white patients [15]. In this study, we aimed to assess the prevalence of CVD risk factors and outcomes in a predominantly Black RA population.

## 2. Methods

In this cross-sectional study, we utilized the International Classification of Diseases, Ninth Revision, Clinical Modification (ICD-9-CM 714.0) and the International Classification of Diseases, Tenth Revision (ICD-10-CM M06.00–M06.09), to identify RA as either the principal or secondary diagnosis in the discharge summary. We included all inpatient discharges, from 1 January 2010 to 30 May 2017, that took place at State University of New York Downstate Medical Center-University Hospital of Brooklyn and New York City (NYC) Health + Hospitals/Kings County, which serve the population of Central Brooklyn, NY. Prior to the initiation of the study, the protocol was approved by the SUNY Downstate Institutional Review Board and the Office of Research Administration for implementation at NYC Health + Hospitals/Kings County. We selected patients, 18 years or older by 1 January 2010 to be included in the study. Two investigators (IMM, SYZ) independently reviewed the cases identified by ICD-codes to confirm RA diagnosis. We used physician entries (inpatient/outpatient notes and consultations) and the presence of disease-modifying anti-rheumatic drugs (DMARDs) in the medication reconciliation or DMARDs prescriptions. Data abstraction was performed for confirmed cases utilizing the study data collection sheet. Demographics and clinical data including history of smoking, year of RA-diagnosis (according to 2010 American College of Rheumatology criteria), comorbidities, laboratory data, hand imaging and treatment regimens were abstracted [15].

A musculoskeletal radiologist (SK) evaluated the bilateral hand radiographs utilizing the Simple Erosion Narrowing Score (SENS) to ascertain the presence and number of erosions and joint space narrowing [16]. The radiologist was blinded to the serologic status of the reviewed cases.

Descriptive statistics using IBM ® SPSS version 23 (name, city, state, country) was applied. Measures of central tendency and dispersion for continuous variables, and frequency distribution for categorical variables were used. Data is presented as the mean ± standard error of the mean (±SEM). We compared our predominantly Black RA population with the predominantly White cohort of the Consortium of Rheumatology Researchers of North America (CORRONA) to assess differences in CVD risk profiles, CVD outcomes, features of RA disease severity, and therapeutic patterns including the use of steroids, DMARDs and biologics [7]. We used a *t*-test to compare between groups for continuous variables, and Chi square analysis for categorical ones.

## 3. Results

**Figure 1:**
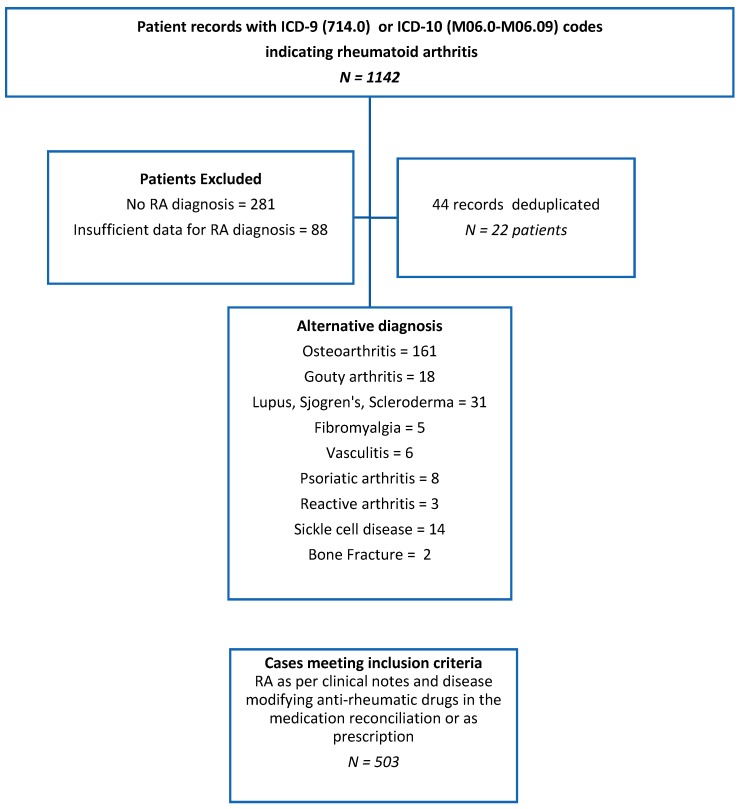
Flow chart delineating the selection procedure of the rheumatoid arthritis cases in the study.ICD-9: International Classification of Diseases, Ninth Revision, Clinical Modification, ICD-10: International Classification of Diseases, Tenth Revision, Clinical Modification.

Of the 1142 RA patients identified by ICD codes, 281 had no clinical documentation to support RA diagnosis, 88 had insufficient data for confirmation of RA diagnosis, and 248 had an alternative diagnosis. Forty-four records were identified as duplicates and abstracted as 22 unique patients. This process resulted in the identification of 503 patients with confirmed RA that were included in this analysis (Figure 1). The mean disease duration (in years) was 13.1 ± 0.7, and mean patients’ age was 64.7 ± 0.6 (±SEM). Of them, 87.9% were women, 88.5% were Black and 9.2% Hispanics. Mean body mass index (BMI) was 28.92 ± 0.36 with 37.2% of the patients having a BMI ≥ 30 (Kg/m^2^). Women were significantly older compared to men, with a mean age of 65 ± 0.67 vs. 61 ± 2.19 respectively (*p* < 0.04) (Table 1).

The rates of traditional CVD risk factors in our study cohort (predominantly Black) vs. the CORRONA study cohort (predominantly White) respectively were: hypertension (66.6% vs. 29%), dyslipidemia (41.3% vs. 25%), diabetes (28.5% vs. 8%), and smoking (29.4% vs. 34%) (Table 1).

CVD risk factors were not significantly different between men and women in our cohort (Table 2). At least one traditional CVD risk factor (BMI ≥ 30, positive smoking history, dyslipidemia, diabetes or hypertension) was encountered in 87.4% of the RA cohort with the presence of three or more traditional risk factors occurring in 37%. Rheumatoid arthritis-specific risk factors (RA disease more than 10 years, presence of joint erosions, joint space narrowing, extra-articular manifestations, BMI < 20, Rheumatoid Factor (RF) or anti-citrullinated peptide antibodies (ACPA) positivity, erythrocyte sedimentation rate (ESR) ≥ 42 mm/h. or C-reactive protein (CRP) ≥ 10 mg/L) were seen in 58% of the cases (Table 3).

The examined CVD outcomes included myocardial infarction (MI) or known coronary artery disease (CAD) (19.8%), which were similar to those reported in the CORRONA study [7]. The rates of other CVDs in our cohort that were not reported in the CORRONA study were: congestive heart failure (14.8%), stroke or transient ischemic attack (10.1%) and atrial fibrillation (8.4%). For ESR, the mean was 62.7 ± 2.1 mm/h., CRP was 48.7 ± 4.2 and CRP > 4 mg/L was found in 74.6% of our cohort. 86.6% of our patients were either RF or ACPA positive (compared to 77% in the CORRONA study), and dual RF-ACPA positivity was found in 54%. We also compared the rates of traditional CVD risk factors, CV outcomes and RA-specific risk factors among the seropositive and seronegative groups; statistical significance was found for the frequency of RA-specific risk factors (89.5% vs. 67.7%) and ESR ≥ 42 mm/h. (70% vs. 38.4%) *p* < 0.001 (Table 3).

Utilizing SENS scoring for hand radiographs, periarticular osteopenia, joint space narrowing and joint erosions were observed in 95.2%, 69% and 66.5% respectively of our RA patients, while joint erosions were reported in 50.7% of the cohort in the 2010 CORRONA study [19]. Our patients’ mean number of joint erosions was 10.73 ± 0.98 (maximum = 32) and the mean number of joint space narrowing was 17 ± 1.05 (maximum = 30). No statistical significance was noted among the seropositive and seronegative groups for hand imaging findings. We recorded glucocorticoid use in 56% of our patients (vs. 30% for CORRONA) with an average dose of 8.1 ± 0.95 mg/day, nonsteroidal anti-inflammatory drugs (NSAIDs) use in 22% and narcotics in 8.1%. With regard to DMARDs use, 40.3% of the patients were on Methotrexate with an average dose of 6.6 ± 0.47 mg/week, other DMARDs in 43% and biologics in 16.2% (Table 4). Our data also demonstrates a higher CV burden and higher CV outcomes among steroid users in contrast with the lower rates of CV outcomes in non-steroid users treated with DMARDs and biologics (Table 5).

## 4. Discussion

Our predominantly Black RA population had higher rates of CVD risk factors compared to predominantly White RA cohorts of previous studies. These CVD risk factors included obesity, hypertension, dyslipidemia and diabetes. The CORRONA is a US-based registry of about 25,000 RA patients which is followed longitudinally [7]. RA disease activity and its impact on cardiovascular (CV) end points have been the subject of a number of publications by the CORRONA investigators [6,7,19,20]. Eighty-nine percent of the patients in the CORRONA registry are White, contrasting with our inner-city RA population of 88.5% Black and 9.2%, Hispanics.

Similarities between our study cohort and the CORRONA cohort include female predominance and nearly identical BMI (28.9 vs. 29.2). However, our population differs from the CORRONA patients in a number of characteristics. Lower rates of current smokers and ever smokers were found among our patients, i.e., 11.5% and 29.5% respectively (vs. 19% and 37% in CORRONA respectively), and higher rates of hypertension, dyslipidemia and diabetes. Eighty-seven percent of our patients have at least one traditional CVD risk factor present, with more than a third of them carrying three or more traditional risk factors, which supports the notion that our population is at a higher risk for CVD events and mortality.

Our RA population was also found to have a higher frequency of seropositivity, as demonstrated by the presence of rheumatoid factor or anti-citrullinated antibodies (85.8% vs. 77% in CORRONA), and more than half of our patients had had RA disease for longer than 10 years. Our study includes radiological evaluation with evidence of joint damage (e.g., joint erosions and joint space narrowing) as markers of disease severity. Furthermore, our study examined the presence of extra-articular manifestations of RA (e.g., vasculitis, ocular involvement, subcutaneous nodules and interstitial lung disease), and recorded 7.5% of patients with a BMI < 20 (rheumatoid cachexia). The aggressive nature of RA disease seen in our cohort is further supported by the frequency of elevated inflammatory markers such as CRP and ESR. We also found a statistically-significant difference between the seropositive and seronegative RA groups in the rates of RA-specific CVD risk factors, with seropositive patients exhibiting higher prevalence of joint erosions, joint space narrowing, extra-articular disease and elevated serum markers. Seronegative patients demonstrated higher rates of hypertension compared with the seropositive group (66.6% vs. 50.4% respectively); however, this finding was not statistically significant, likely due to the small sample size. Furthermore, our data demonstrates a higher burden of both traditional and RA-specific risk factors in our study cohort as compared with the CORRONA cohort [7]. Interestingly, the rate of CAD was similar (19.8%) to the one observed in the 2015 CORRONA study (17%), although a higher rate of CAD or MI amongst our patients is to be expected, given the high inflammatory burden. However, earlier data from the CORRONA cohort [19] revealed that only 8.5% of the patients had CAD at baseline, indicating that our cross-sectional study has captured higher rates of CAD in our population.

Increased risk for CVD events and mortality in RA has been the subject of several meta-analyses, population studies and mechanistic experiments [4,21,22]). Chronic inflammation contributes to atherosclerosis and CVD. Serum markers have also been found to be independent predictors of CAD and ischemic strokes [14,17,18]. Elevated CRP portends higher risk for MI, heart failure, atherosclerosis and mortality among RA patients [23,24]. In addition, high ESR has been associated with CVD risk. ESR > 42 mm/h. was shown to be linked to MI and stroke, with levels above 50 mm/hr. increasing 10-fold the risk for CVD [18]. Furthermore, efforts to develop a reliable RA CVD risk score have resulted in overestimates or underestimates of CVD risk in RA population [14].

RA patients are prone to a specific form of dyslipidemia which is characterized by high levels of triglycerides, low levels of both low-density lipoproteins (LDL) and high density lipoproteinemia. This phenomenon is called “lipid paradox” given that there is increased CV risk despite low levels of lipoproteins. High-density lipoprotein appears to be dysfunctional in RA patients, leading to inadequate clearance of lipids from the atheromatous plaque formation. This pro-inflammatory high-density lipoprotein positively correlates with elevated acute phase reactants and disease activity [25,26,27,28]. We can postulate that in our population, active RA disease and elevated inflammatory markers contribute to the high prevalence of the observed traditional CVD risk factors.

The obesity rates observed in our population could be explained by decreased physical activity secondary to active RA disease, which further contributes to the burden of traditional CVD risk. On the other hand, “obesity paradox” is also observed in RA, with BMI < 20 Kg/m^2^ correlating with high disease activity and higher BMI reflecting better disease control [14].

Additionally*,* the higher use of prednisone among our patients, administered for disease control, adds to the CVD risk [29]. Methotrexate, DMARDs and biologics administered to manage RA have been demonstrated to reduce CV risk among RA patients given their effect on reducing chronic inflammation [14]. In contrast, in our patient population, the utilization rates of methotrexate, DMARDs and biologics were found to be lower than those observed in the CORRONA registry.

Finally, our study is limited by the retrospective nature of the analysis, lack of available RA-specific disease activity measurements, characterization of cardiac involvement, stroke type (ischemic vs. hemorrhagic), response to therapeutic interventions and survival outcomes. Inaccuracy in coding explains the number of cases in which RA diagnosis was not found in the clinical documentation. There is also data missing at random, which we believe is harmless and does not represent a systematic bias. We plan, however, to undertake a longitudinal study to assess disease activity at presentation and at follow-up intervals, disease complications including CVD events, response to primary and secondary prevention of traditional and RA specific risk factors and outcomes. We also acknowledge the limitation of the gender comparison made in Table 2, given the limited number of men in RA, a predominantly female disorder.

## 5. Conclusions

This is the first study to evaluate CVD risk in a predominately Black RA population that included radiological as well as serological assessment of disease severity, together with characterization of therapeutic patterns. We observed higher rates of traditional CVD risk factors, including obesity, diabetes, hypertension, dyslipidemia, compared to the White RA cohort. Our population had more aggressive disease with higher rates of seropositivity, joint narrowing/erosions and elevated inflammatory markers. The combination of higher rates of traditional and RA-specific risk factors confers on our patients a high risk for CVD events. Our RA population characteristics require therapeutic interventions to address disease control and targeted management of comorbidities that involve revised risk stratification aiming at reducing CVD morbidity and mortality in this highly vulnerable population.

## Figures and Tables

**Table 1:** Population characteristics.

**Total Number of Patients: 503** **Mean Age 64.76 ± 0.66 (±SEM)**	
Number of women	87.9%
Number of men	12.1%
Women’s mean age (±SEM) years	65 ± 0.68
All patients age in years (±SEM)	64.7 ± 0.6
**Race/Ethnicity**	
White	7.2%
Black	88.5%
Native American	0.2%
Asian Pacific Islander	0.6%
Hispanics	9.2%
**Other Characteristics**	
Non-smoker	70.6%
Ever smoker	29.4%
BMI (mean ± SEM)	28.9 ± 0.36
BMI ≥ 30 Kg/m^2^	37.2%
Diabetes mellitus	28.5%
Hypertension	66.6%
Hyperlipidemia	41.3%
CAD or AMI	19.8%
Prior cerebrovascular accident	10.1%
Atrial fibrillation	8.4%
Congestive heart failure	14.8%

Percentages were calculated based on the number of patients with data available on the measure. CAD: coronary artery disease; MI: myocardial infarction, BMI: body mass index, SEM: standard error of the mean.

**Table 2:** Gender comparison.

	Women (*n* = 442)	Men (*n* = 61)
Age in years (mean ± SEM)	65.2±0.6	61 ± 2.1
Ever smoker (current/past)	(28.2%)	(37.9%)
BMI Kg/m^2^ (mean ± SEM)	29.9 ± 0.382	27.51 ± 0.924
BMI >30 Kg/m^2^	157/404 (38.9%)	12/50 (24%)
Diabetes mellitus	121/419 (28.9%	15/58 (25.9%)
Hypertension	289/429 (67.4%)	36/59 (61%)
Hyperlipidemia	172/406 (42.4%)	20/59 (33.9%)
CAD or MI	77/390 (19.7%)	11/55 (20%)
Prior cerebrovascular accident	42/398 (10.6%)	4/57 (7%)
Atrial fibrillation	35/409 (8.6%)	4/56 (7.1%)
Congestive heart failure	65/417 (15.6%)	5/57 (8.8%)

Percentages were calculated based on the number of patients with data available on the measure.

**Table 3:** Cardiovascular risk factors and outcomes considering the serotype.

	All Patients	Seropositive RF+ and/or ACPA+ (*n* = 201)	Seronegative RF- and ACPA- (*n* = 31)	*p*-Value
**Cardiovascular risk factors**				
Hypertension (HTN)	325/488 (66.6%)	65/129 (50.4%)	20/30 (66.6%)	0.98
Hyperlipidemia (HLP)	192/465 (41.3%)	77/185 (41.6%)	15/31 (48.4%)	0.48
Diabetes mellitus (DM)	136/477 (28.5%)	28/175 (16%)	4/29 (13.7%)	0.76
**Traditional risk factors for CVD**				
Any traditional CVD risk factor present	436/499 (87.4%)	175/199(87.9%)	29/31(93.5%)	0.35
≥3 traditional risk factors	157/425 (37%)	67/194 (34.5%)	7/29 (24.3%)	0.27
**Rheumatoid arthritis-specific risk factors**				
RA-specific risk factors for CVD	292/503 (58%)	180/201 (89.5%)	21/31 (67.7)	0.001 *
RA duration of disease >10 years	108/197 (54.8%)	61/114 (53.5%)	11/16 (68.5%)	0.25
BMI < 20 Kg/m^2^	34/454 (7.5%)	16/189 (8.4%)	1/27 (3.7%)	0.39
Joint erosions	125/188 (66.5%)	82/120 (68.3%)	6/13 (46.15%)	0.11
Joint space narrowing	130/188 (69.1%)	84/120 (70%)	7/13 (53.8%)	0.23
Extra-articular disease	38/503 (7.5%)	12/201 (6.9%)	0/31 (0%)	0.13
CRP > 10 mg/L **	180/285(63.1%)	102/171(56.9%)	12/24 (50%)	0.36
ESR > 42 mm/h ***	198/313(63.1%)	124/177 (70%)	10/26 (38.4%)	0.001*
Positive Rheumatoid Factor (RF)	186/247(75.3%)	186/247 (75.3%)	0%	NA
Positive Anti-citrullinated ab. (ACPA)	124/178(69.6%)	124/178 (69.6%)	0%	NA
Either RF+ or ACPA+	201/232 (86.6%)	201/232 (86.6%)	0%	NA
Double seropositive (RF+ and ACPA+)	109/201(54%)	109/201(54%)	0%	NA
**Cardiovascular outcomes**				
Congestive heart failure	70/474 (14.8%)	27/188 (14.3%)	2/30 (6.6%)	0.25
CAD or MI	88/445 (19.8%)	28/175 (16%)	4/29 (13.8%)	0.76
Prior cerebrovascular accident (CVA)	46/455 (10.1%)	18/183 (9.8%)	3/29 (10.3%)	0.93
Atrial fibrillation	39/465 (8.4%)	14/187 (7.4%)	1/30 (3.3%)	0.41
Any CVD outcome	175/503 (34.8%)	59/201 (29.4%)	10/31 (32.3%)	0.29

Percentages were calculated based on the number of patients with data available on the measure. RF: rheumatoid factor, ACPA: anti-citrullinated peptide antibody, ESR: erythrocyte sedimentation rate, CRP: C reactive protein. * *p* < 0.01; ** C reactive protein > 10 mg/L. is associated with MI [17] *** Erythrocyte sedimentation rate >42 mm/h. is associated with MI and ischemic stroke risk [17,18].

**Table 4:** Therapeutic Management.

Glucocorticoids	238/425 (56%)
PDN mean daily dose (±SEM) in mg.	8.14 ± 0.95
NSAIDs	89/406 (22.1%)
Narcotics	33/406 (8.1%)
Methotrexate (MTX)	175/434 (40.3%)
MTX mean weekly dose (±SEM) in mg.	6.6 ± 0.47
Other DMARDs	153/356 (43%)
Biologics	68/420 (16.2%)

PDN: Prednisone, NSAID: nonsteroidal anti-inflammatory drugs, DMARD: disease-modifying, anti-rheumatic drugs.

**Table 5:** Therapeutic regimens and associated CVD risk factors and outcomes.

	Only Steroids	Steroids and DMARDs/Biologics	Only DMARDs/Biologics
Any CVD risk factor	205/436 (47%)	152/436 (34.9%)	119/436 (27.3%)
≥3 CVD risk factors	69/157 (44%)	52/157 (33.1%)	46/157 (29.3%)
Any CVD outcome	81/175 (46.3%)	62/175 (35.4%)	45/175 (25.7%)
